# Supplementary material for: Bayesian refinement of protein structures and ensembles against SAXS data using molecular dynamics
Source: PLoS Comput Biol. 2017 Oct 18;13(10):e1005800. doi: 10.1371/journal.pcbi.1005800 (PMC5662244; doi:10.1371/journal.pcbi.1005800)
Supplement: S1 Archive — (BZ2) [file pcbi.1005800.s011.tar.bz2 › gromacs_4.62_bayesian_saxs_refi/share/html/online/cpt.html]

cpt file format


## cpt file format

|  |  |
| --- | --- |
| Main Table of Contents | **VERSION 4.6** |
| GROMACS homepage | **Sat 19 Jan 2013** |

---

### Description

The cpt file extension stands for portable checkpoint file.
The complete state of the simulation is stored in the checkpoint file,
including extended thermostat/barostat variables, random number states
and NMR time averaged data.
With domain decomposition also the some decomposition setup information
is stored.

See also mdrun.

---

http://www.gromacs.org
